# Uropathogens and prognosis among patients with hospital-diagnosed acute pyelonephritis: insights from a 19-year population-based cohort study

**DOI:** 10.1007/s15010-026-02729-7

**Published:** 2026-01-23

**Authors:** Lise Skovgaard Svingel, Mette Nørgaard, Christian Fynbo Christiansen, Henrik Birn, Hans Linde Nielsen, Kirstine Kobberøe Søgaard

**Affiliations:** 1 Department of Clinical Epidemiology, Department of Clinical Medicine, Aarhus University and Aarhus University Hospital, Olof Palmes Allé 43-45, 8200 Aarhus N, Denmark; 2 Departments of Clinical Medicine and Renal Medicine, Aarhus University and Aarhus University Hospital, Aarhus, Denmark; 3 Department of Clinical Microbiology, Aalborg University Hospital, Aalborg, Denmark; 4 Department of Clinical Medicine, Aalborg University, Aalborg, Denmark

**Keywords:** Epidemiology, Population-based study, Pyelonephritis, Urinary tract infections, Aetiology, Escherichia coli

## Abstract

**Purpose:**

The microbial aetiologies of acute pyelonephritis (APN) may change over time. We aimed to describe long-term trends in microbiological diagnostics and pathogen distribution in patients with hospital-diagnosed APN, and to characterise clinical outcomes by pathogens.

**Methods:**

We conducted a population-based, serial cross-sectional and cohort study of patients with hospital-diagnosed APN in North Denmark across three periods covering 2000-2018. National health registries were linked with microbiological data to describe temporal trends in microbiological diagnostics and pathogen distribution, and to provide a descriptive comparison of median length of stay (LOS) with interquartile range (IQR) and 30-day cumulative mortality with 95% confidence interval (CI) between *Escherichia coli* and non-*E. coli* APN.

**Results:**

We identified 5338 APN episodes among 4773 patients. The proportion with urine culture increased from 75.1% in 2000-2006 to 92.9% in 2013-2018, with a concomitant increase in the proportion with a positive urine culture from 44.4% to 56.7%. The median LOS declined by 2 days across calendar periods. *E. coli* remained the predominant pathogen with a prevalence in the range 77.3%-81.9%. Non-*E. coli* APN was more common in male, older, and comorbid patients, and was characterised by longer LOS (median 5 days [IQR: 3-8] vs. 4 days [IQR: 2-6]) and higher 30-day mortality (3.7% [95% CI 2.3%-5.2%] vs. 1.0% [95% CI 0.6%-1.5%]) compared with *E. coli*.

**Conclusion:**

Microbiological testing increased during the study period, and the pathogen distribution remained largely stable with *E. coli* as the predominant uropathogen. Non-*E. coli* infections were associated with slightly less favourable short-term outcomes.

**Supplementary Information:**

The online version contains supplementary material available at 10.1007/s15010-026-02729-7.

## Introduction

In recent years, the incidence of acute pyelonephritis (APN) has increased in hospital settings [1–4]. This severe kidney infection is typically caused by Gram-negative bacteria, particularly *Escherichia coli*, and the initial treatment relies on empirical antibiotic therapy tailored to local resistance patterns and patient-specific risk factors [5, 6]. Given that prompt and appropriate treatment of infections has been suggested to improve treatment effectiveness and clinical outcomes such as length of stay (LOS) and mortality [7–9], the increasing prevalence of antimicrobial resistance, including to third-generation cephalosporins, poses a growing global challenge [10].

The epidemiology of APN may be influenced by temporal changes in healthcare practices, such as greater use of urinary catheters and interventional urological procedures, along with shifts in population demographics. An increased number of individuals of advanced age living with multiple chronic conditions may be at heightened risk of severe disease and infection with atypical or resistant pathogens [5, 11, 12]. Together, these factors highlight the need to better understand microbial trends, diagnostic approaches, and outcomes among patients with APN, to inform management strategies.

This study aimed to describe 19-year trends in microbiological diagnostics and microbial aetiology in patients with hospital-diagnosed APN and to characterise clinical outcomes according to the identified pathogens.

## Materials and Methods

### Design and setting

We conducted a descriptive, registry-based study in North Denmark from 2000 to 2018, employing both serial cross-sectional and cohort designs. For descriptive analyses, the 19-year study period was divided into three calendar periods (2000–2006, 2007–2012, and 2013–2018). The selection of these intervals was guided by previously documented changes in the incidence of hospital-diagnosed APN in this population[3] and by the regional administrative reform in 2007, which altered the size of the catchment area population (from approximately 494,000 to 590,000 residents) [13, 14]. These periods provided relatively stable population denominators and ensured sufficient sample size for stratified analyses.

Denmark’s tax-funded healthcare system provides free access to general practitioners and public hospitals for all residents. Throughout the study period, the Department of Clinical Microbiology at Aalborg University Hospital was the sole provider of microbiological diagnostics for the study region with no private or alternative suppliers [15].

Guidelines recommended urine cultures with antimicrobial susceptibility testing (AST) for all suspected cases of APN to support diagnosis and therapy adjustments [5, 16, 17]. While Denmark is considered a low antimicrobial resistance setting, the national surveillance programme reported gradually increasing urinary resistance rates to third-generation cephalosporins in *E. coli* during the study period, primarily caused by extended-spectrum beta-lactamases (ESBLs) [18]. Pivmecillinam was commonly used as empirical antibiotic therapy for outpatient treatment of APN in adults, and intravenous mecillinam was frequently applied in inpatient settings based on local clinical practice and susceptibility patterns.

### Study population and variables

Supplementary Tables 1 and 2 present the data sources and codes used in this study. National health registries were unambiguously linked with microbiological data using unique 10-digit personal identifiers. From this population, we included all patients diagnosed with APN during inpatient admissions or visits to acute hospital settings. Multiple APN diagnoses within 30 days were considered part of the same episode, with index date defined by the first admission date. We excluded patients with a prior diagnosis of chronic pyelonephritis (from 1994 to the index date).

Supplementary Table 1 describes the microbiological methods applied. For all APN episodes, we identified the results of urine and blood cultures obtained from 30 days before to 4 days after the index date. Descriptive variables included sex and age group (0–2, 3–14, 15–29, 30–49, 50–74, and ≥75 years). Baseline comorbidities, identified from the earliest available record up to the index date, included hypertension, diabetes mellitus, and various uropathies: congenital anomalies of the kidney and urinary tract (CAKUT), acquired obstructive and reflux uropathy, urolithiasis, urinary tract cancer, other kidney diseases, and other urinary tract diseases. For patients under 15 years, only CAKUT was assessed. Additional variables included urinary tract imaging and recent (within 30 days prior to the index date) inpatient admission, interventional urological procedure, and outpatient antibiotic treatment of UTI.

### Statistical analysis

We characterised patients with APN episodes using frequency with column percentages (presented in tables) and row percentages (presented in figures) for categorical variables, and median with interquartile range (IQR) for continuous variables.

**Fig. 1:**
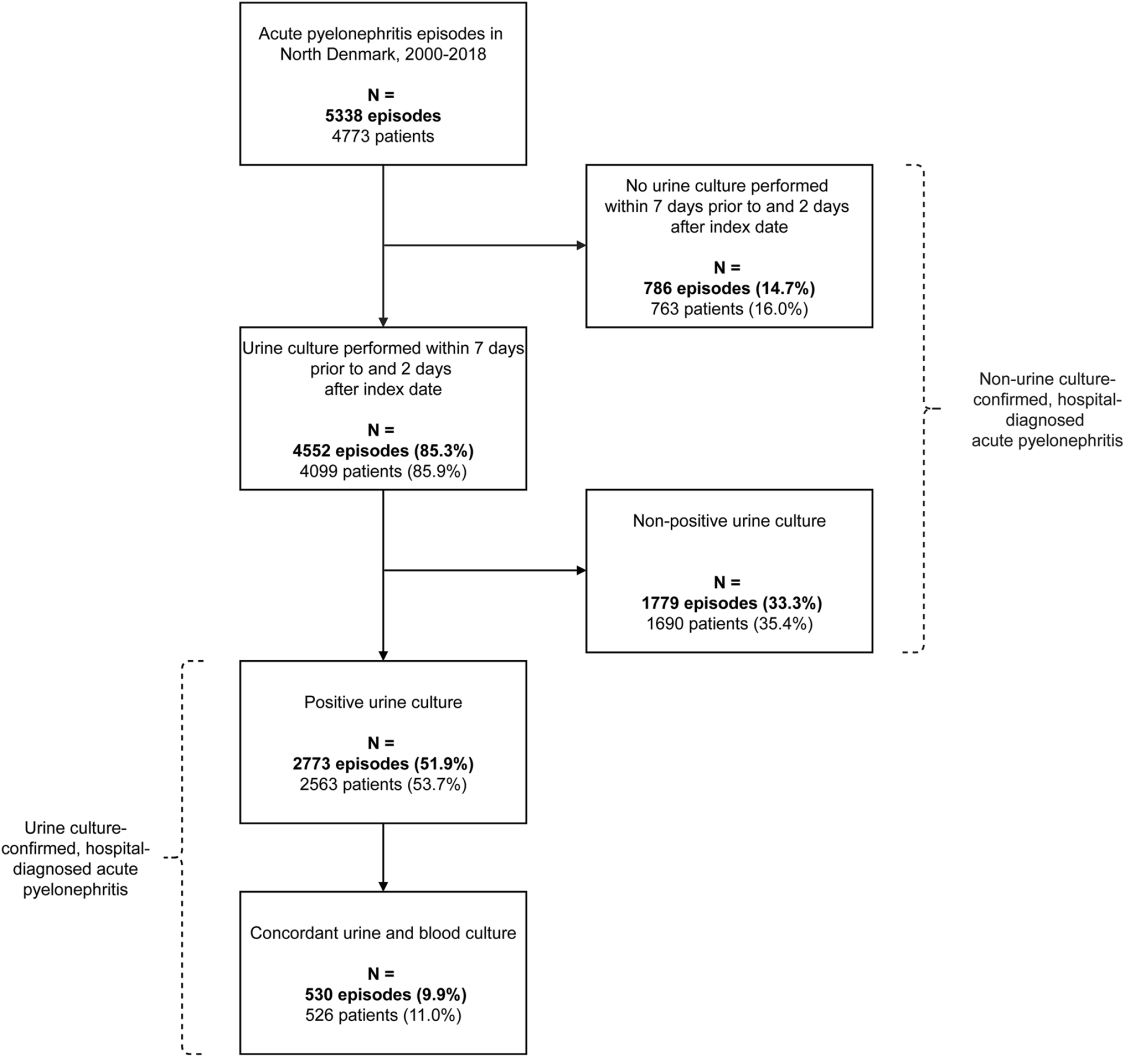
Flowchart illustrating the construction of subgroups of patients with hospital-diagnosed acute pyelonephritis in the primary analysis. Urine cultures were classified as positive following the criteria in Supplementary Table 3. Cultures were classified as non-positive if the specimen was collected using an unreliable method (*e.g.*, bottle or bedpan), the bacterial concentration was below the predefined cut-off, or the culture was deemed contaminated (*i.e.*, containing more than two species and not accompanied by a positive blood culture identifying the same species)

For the primary analysis, we included urine culture together with blood cultures and urinary tract imaging performed from 7 days before to 2 days after the index date. This time window was based on existing literature [11, 19] and the typical clinical course of APN, to ensure inclusion of tests obtained during the pre-hospital diagnostic workup and around the time of admission, while minimizing the inclusion of cultures potentially reflecting hospital-acquired infections. We evaluated urine cultures based on the criteria presented in Supplementary Table 3, using a cut-off concentration of ≥10^3^ colony forming units (CFU)/mL to define significant bacteriuria [16], with the aim of enhancing sensitivity in this hospitalised cohort including patients potentially receiving antibiotics [17]. We assessed temporal trends in microbiological diagnostics across three calendar periods (2000–2006, 2007–2012, and 2013–2018) by reporting the proportion of urine culture-investigated episodes and the proportion of urine culture-positive episodes. Subgroups are defined in Fig. 1. Microbial aetiology was reported as the overall and period-specific prevalence of uropathogens isolated from positive urine cultures. To ensure sufficient numbers to describe temporal trends in patient characteristics by species, we categorized uropathogens as *E. coli* and non-*E. coli* species. Cell counts below five, and any cells from which such small counts could be inferred, were masked to prevent potential identification of individuals and thereby maintain data confidentiality. Finally, clinical outcomes were explored by species and reported both overall and by calendar period. LOS was defined as the duration from first hospital admission to final discharge within the episode and summarised as the median with IQR and 95% CI. Thirty-day mortality was reported as frequencies with percentages and cumulative mortality with 95% CI.

In supplementary analyses, we assessed the robustness of the results using a ≥10^5^ CFU/mL cut-off concentration to define significant bacteriuria. Moreover, two sensitivity analyses evaluated expanded time windows for urine culture inclusion: (1) from 14 days before to 4 days after, and (2) from 30 days before to 4 days after the index date. In both analyses, blood cultures and urinary tract imaging were considered APN-related if performed from 7 days before to 4 days after the index date.

Analyses were performed using R version 4.1.3 (R Foundation for Statistical Computing, Vienna, Austria).

## Results

### Microbiological diagnostics

From 2000 to 2018, we identified 5338 hospital-diagnosed APN episodes among 4773 patients (Fig. 1). In 85.3% of episodes, urine was cultured within 7 days before to 2 days after the index date, and the diagnosis was confirmed by a positive urine culture in 51.9% of episodes. An overall 33.3% of all included patients had received antibiotics prior to urine sampling (Table 1), and this proportion reached 44.9% among patients with non-positive urine cultures (Supplementary Table 4). Additional subgroup patient characteristics are presented in Tables 1, 2, and Supplementary Table 4.

**Table 1:** Baseline characteristics of patients with hospital-diagnosed acute pyelonephritis, stratified by test characteristics and calendar period

	All episodes, n (%)				Episodes with urine culture, n (%)			
	Overall	Calendar period^a^			Overall	Calendar period^a^		
		2000–2006	2007–2012	2013–2018		2000–2006	2007–2012	2013–2018
Total	5338 (100.0)	1330 (100)	1740 (100.0)	2268 (100.0)	4552 (100.0)	999 (100.0)	1446 (100.0)	2107 (100.0)
Sex								
Female	3909 (73.2)	942 (70.8)	1272 (73.1)	1695 (74.7)	3354 (73.7)	713 (71.4)	1066 (73.7)	1575 (74.8)
Male	1429 (26.8)	388 (29.2)	468 (26.9)	573 (25.3)	1198 (26.3)	286 (28.6)	380 (26.3)	532 (25.2)
Age in years								
Median (IQR)	38 (18-66)	42 (21-71)	35 (15-64)	39 (19-66)	37 (17-65)	39 (18-68)	33 (9-62)	38 (18-65)
0-2	565 (10.6)	109 (8.2)	216 (12.4)	240 (10.6)	538 (11.8)	101 (10.1)	203 (14.0)	234 (11.1)
3-14	538 (10.1)	126 (9.5)	206 (11.8)	206 (9.1)	507 (11.1)	113 (11.3)	198 (13.7)	196 (9.3)
15-29	1121 (21.0)	265 (19.9)	356 (20.5)	500 (22.0)	926 (20.3)	183 (18.3)	285 (19.7)	458 (21.7)
30-49	933 (17.5)	250 (18.8)	301 (17.3)	382 (16.8)	789 (17.3)	199 (19.9)	235 (16.3)	355 (16.8)
50-74	1321 (24.7)	302 (22.7)	410 (23.6)	609 (26.9)	1127 (24.8)	221 (22.1)	338 (23.4)	568 (27.0)
≥75	860 (16.1)	278 (20.9)	251 (14.4)	331 (14.6)	665 (14.6)	182 (18.2)	187 (12.9)	296 (14.0)
Comorbidity in age <15 years								
CAKUT	25 (2.3)	9 (3.8)	9 (2.1)	7 (1.6)	≤25	≤10	9 (2.2)	6 (1.4)
Comorbidity in age ≥15 years								
Hypertension	927 (21.9)	252 (23.0)	287 (21.8)	388 (21.3)	767 (21.9)	179 (22.8)	225 (21.5)	363 (21.6)
Diabetes mellitus	688 (16.2)	157 (14.3)	205 (15.6)	326 (17.9)	572 (16.3)	116 (14.8)	155 (14.8)	301 (17.9)
Uropathies								
CAKUT	163 (3.8)	40 (3.7)	56 (4.2)	67 (3.7)	140 (4.0)	29 (3.7)	48 (4.6)	63 (3.8)
Acquired obstructive and reflux uropathy	300 (7.1)	79 (7.2)	96 (7.3)	125 (6.9)	245 (7.0)	50 (6.4)	80 (7.7)	115 (6.9)
Urolithiasis	241 (5.7)	58 (5.3)	81 (6.1)	102 (5.6)	201 (5.7)	41 (5.2)	66 (6.3)	94 (5.6)
Urinary tract cancer	65 (1.5)	17 (1.6)	22 (1.7)	26 (1.4)	50 (1.4)	≤15	15 (1.4)	≤25
Other kidney disease	207 (4.9)	59 (5.4)	60 (4.6)	88 (4.8)	173 (4.9)	43 (5.5)	50 (4.8)	80 (4.8)
Other urinary tract disease	448 (10.6)	120 (11.0)	130 (9.9)	198 (10.9)	374 (10.7)	91 (11.6)	105 (10.0)	178 (10.6)
Recent events^b^								
Inpatient admission	1377 (25.8)	344 (25.9)	443 (25.5)	590 (26.0)	1166 (25.6)	259 (25.9)	356 (24.6)	551 (26.2)
Urological procedure	173 (3.2)	43 (3.2)	63 (3.6)	67 (3.0)	147 (3.2)	32 (3.2)	53 (3.7)	62 (2.9)
Antibiotics	1778 (33.3)	473 (35.6)	567 (32.6)	738 (32.5)	1546 (34.0)	370 (37.0)	474 (32.8)	702 (33.3)
Additional tests^c^								
Blood culture	3989 (74.7)	853 (64.1)	1305 (75.0)	1831 (80.7)	3721 (81.7)	782 (78.3)	1199 (82.9)	1,40 (82.6)
Imaging	4142 (77.6)	1034 (77.7)	1363 (78.3)	1745 (76.9)	3529 (77.5)	776 (77.7)	1134 (78.4)	1619 (76.8)

^a^The catchment population increased from approximately 494,000 in the year 2000 to approximately 590,000 in the year 2018

^b^Within 30 days prior to the index date

^c^From 7 days before to 2 days after the index date

*CAKUT* congenital anomalies of the kidney and urinary tract; *IQR* interquartile range

**Table 2:** Baseline characteristics of patients with urine culture-confirmed, hospital-diagnosed acute pyelonephritis, stratified by test characteristics and calendar period

	Episodes with positive urine culture^a^, n (%)				Episodes with concordant urine and blood culture^a^, n (%)			
	Overall	Calendar period^b^			Overall	Calendar period^b^		
		2000-2006	2007-2012	2013-2018		2000-2006	2007-2012	2013-2018
Total	2773 (100.0)	591 (100.0)	895 (100.0)	1287 (100.0)	530 (100.0)	151 (100.0)	164 (100.0)	215 (100.0)
Sex								
Female	2058 (74.2)	423 (71.6)	651 (72.7)	984 (76.5)	395 (74.5)	108 (71.5)	128 (78.0)	159 (74.0)
Male	715 (25.8)	168 (28.4)	244 (27.3)	303 (23.5)	135 (25.5)	43 (28.5)	36 (22.0)	56 (26.0)
Age in years								
Median (IQR)	31 (7-64)	39 (12-68)	27 (5-61)	32 (10-64)	62 (41-76)	65 (42-78)	62 (40-72)	62 (40-76)
0-2	458 (16.5)	80 (13.5)	172 (19.2)	206 (16.0)	15 (2.8)	^c^	^c^	^c^
3-14	357 (12.9)	71 (12.0)	145 (16.2)	141 (11.0)	10 (1.9)	^c^	^c^	^c^
15-29	523 (18.9)	96 (16.2)	154 (17.2)	273 (21.2)	56 (10.6)	12 (7.9)	17 (10.4)	27 (12.6)
30-49	409 (14.7)	108 (18.3)	125 (14.0)	176 (13.7)	99 (18.7)	35 (23.2)	30 (18.3)	34 (15.8)
50-74	629 (22.7)	126 (21.3)	186 (20.8)	317 (24.6)	206 (38.9)	49 (32.5)	72 (43.9)	85 (39.5)
≥75	397 (14.3)	110 (18.6)	113 (12.6)	174 (13.5)	144 (27.2)	49 (32.5)	36 (22.0)	59 (27.4)
Comorbidity in age <15 years								
CAKUT	≤20	8 (5.3)	7 (2.2)	≤5	≤5	^c^	^c^	^c^
Comorbidity in age ≥15 years								
Hypertension	418 (21.3)	107 (24.3)	113 (19.6)	198 (21.1)	135 (26.7)	42 (29.0)	43 (27.7)	50 (24.4)
Diabetes mellitus	342 (17.5)	69 (15.)	89 (15.4)	184 (19.6)	106 (21.0)	32 (22.1)	29 (18.7)	45 (22.0)
Uropathies								
CAKUT	85 (4.3)	19 (4.3)	29 (5.0)	37 (3.9)	19 (3.8	^c^	^c^	^c^
Acquired obstructive and reflux uropathy	131 (6.7)	22 (5.0)	40 (6.9)	69 (7.3)	39 (7.7)	9 (6.2)	15 (9.7)	15 (7.3)
Urolithiasis	113 (5.8)	24 (5.5)	34 (5.9)	55 (5.9)	25 (5.0)	10 (6.9)	6 (3.9)	9 (4.4)
Urinary tract cancer	24 (1.2)	7 (1.6)	8 (1.4)	9 (1.0)	8 (1.6)	^c^	^c^	^c^
Other kidney disease	94 (4.8)	25 (5.7)	25 (4.3)	44 (4.7)	34 (6.7)	10 (6.9)	10 (6.5)	14 (6.8)
Other urinary tract disease	213 (10.9)	53 (12.0)	61 (10.6)	99 (10.5)	58 (11.5)	17 (11.7)	19 (12.3)	22 (10.7)
Recent events^d^								
Inpatient admission	734 (26.5)	162 (27.4)	222 (24.8)	350 (27.2)	148 (27.9)	44 (29.1)	45 (27.4)	59 (27.4)
Urological procedure	91 (3.3)	20 (3.4)	37 (4.1)	34 (2.6)	22 (4.2)	7 (4.6)	5 (3.0)	10 (4.7)
Antibiotics	748 (27.0)	178 (30.1)	226 (25.3)	344 (26.7)	125 (23.6)	38 (25.2)	40 (24.4)	47 (21.9)
Additional tests^e^								
Blood culture	2299 (82.9)	472 (79.9)	765 (85.5)	1062 (82.5)	530 (100.0)	151 (100.0)	164 (100.0)	215 (100.0)
Imaging	2139 (77.1)	464 (78.5)	695 (77.7)	980 (76.1)	414 (78.1)	119 (78.8)	134 (81.7)	161 (74.9)

^a^Urine cultures were classified as positive following the criteria in Supplementary Table 3

^b^The catchment population increased from approximately 494,000 in the year 2000 to approximately 590,000 in the year 2018

^c^Not stratified due to small cell counts

^d^Within 30 days prior to the index date

^e^From 7 days before to 2 days after the index date

*CAKUT* Congenital anomalies of the kidney and urinary tract; *IQR* Interquartile range

**Fig. 2:**
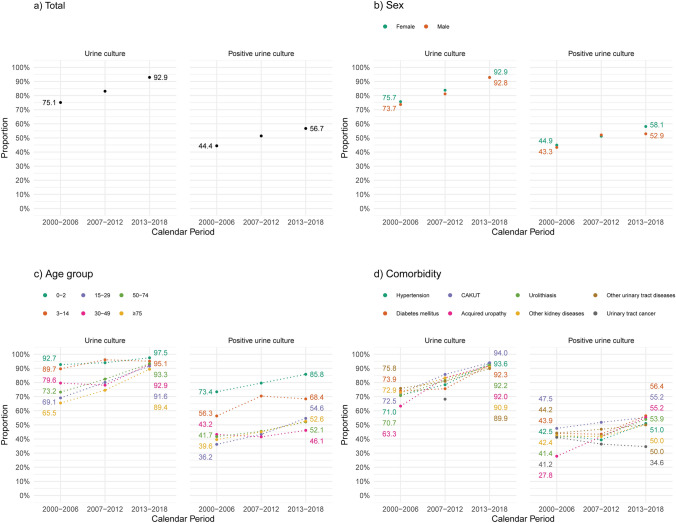
Proportion of APN episodes with urine culture^a^ and positive urine culture^b^ over time, total (**a**) and stratified by sex (**b**), age group (**c**^c^), and comorbidity in age ≥15 years (**d**^c, d^). ^a^ Proportion calculated by dividing the number of episodes with urine culture during a calendar period (presented in Table 1) by the total number of episodes during the same calendar period (presented in Table 1). ^b^ Proportion calculated by dividing the number of episodes with positive urine culture during a calendar period (presented in Table 2) by the total number of episodes during the same calendar period (presented in Table 1). ^c^ Dotted lines serve as visual aid and do not represent interpolated data. Some data points overlap and may therefore not be visually distinguishable. ^d^ To maintain consistency with masking of small cell counts in Table 1, certain proportions for the urinary tract cancer stratum were omitted from the figure. *CAKUT* congenital anomalies of the kidney and urinary tract

Over time, we observed an increase in both use of urine cultures and confirmation by positive urine culture. The proportion of urine culture-investigated episodes rose from 75.1% in 2000-2006 to 92.9% in 2013-2018 (Fig. 2a), primarily due to increased testing in patients aged ≥15 years (Fig. 2c). Concurrently, the proportion of urine culture-positive episodes increased from 44.4% to 56.7%, with the most notable increases observed among female patients and in ages <30 and ≥50 years (Fig. 2a-c). Increasing proportions were also observed across all baseline comorbidities, except urinary tract cancer, and among patients who had recently been admitted to hospital, undergone an interventional urological procedure, or received antibiotics (Fig. 2d, and Tables 1 and 2).

Concomitantly, the use of blood cultures increased from 64.1% of APN episodes in 2000-2006 to 80.7% in 2013-2018 (Table 1), rendering a total of 697 blood culture-positive episodes, hereof 530 (9.9% of all episodes) with concordant urine and blood culture, i.e., yielding the same species (Table 2). In contrast, the proportion of episodes examined by urinary tract imaging remained stable over time (76.9%-78.3%) (Table 1).

### Microbial aetiology

A total of 2849 isolates were detected in 2773 positive urine cultures. *E. coli* (including ESBL-producing strains) was the dominant pathogen, accounting for 79.8% of all isolates (Table 3). Secondary uropathogens comprised 18.3%, with *K. pneumoniae* (including ESBL-producing strains) representing 4.4% of all isolates. Enterococci were less common than the major Gram-negative uropathogens identified in this cohort, accounting for 3.9% of all isolates.

**Table 3:** Microbial species isolated from positive urine culture from patients with hospital-diagnosed acute pyelonephritis, overall and by calendar period

Species category	Species	All isolates, n (%)			
		Overall	Calendar period^a^		
			2000-2006	2007-2012	2013-2018
	Total	2849 (100.0)	609 (100.0)	917 (100.0)	1323 (100.0)
Primary (group I)	All	2305 (80.9)	476 (78.2)	728 (79.4)	1101 (83.2)
	*Escherichia coli*^b^	2220 (77.9)	471 (77.3)	710 (77.4)	1039 (78.5)
	*Escherichia coli*, ESBL^c^	54 (1.9)	0 (0.0)	9 (1.0)	45 (3.4)
	Other^d^	31 (1.1)	5 (0.8)	9 (1.0)	17 (1.3)
Secondary (group II)	All	521 (18.3)	127 (20.9)	179 (19.5)	215 (16.3)
	*Klebsiella pneumoniae*^b^	120 (4.2)	35 (5.7)	42 (4.6)	43 (3.3)
	*Klebsiella pneumoniae*, ESBL^c^	5 (0.2)	0 (0.0)	^e^	^e^
	*Klebsiella oxytoca*	48 (1.7)	10 (1.6)	19 (2.1)	19 (1.4)
	*Klebsiella aerogenes*	5 (0.2)	^e^	^e^	^e^
	*Klebsiella* species, other	9 (0.3)	^e^	^e^	^e^
	*Proteus mirabilis*	35 (1.2)	13 (2.1)	11 (1.2)	11 (0.8)
	*Proteus vulgaris*	9 (0.3)	^e^	^e^	^e^
	*Enterobacter cloacae*	19 (0.7)	^e^	^e^	^e^
	*Citrobacter freundii*	9 (0.3)	^e^	^e^	^e^
	*Citrobacter koseri*	5 (0.2)	^e^	^e^	^e^
	*Morganella morganii*	8 (0.3)	^e^	^e^	^e^
	*Serratia marcescens*	5 (0.2)	^e^	^e^	^e^
	Enterobacterales, other^f^	17 (0.6)	^e^	^e^	^e^
	*Pseudomonas aeruginosa*	69 (2.4)	20 (3.3)	24 (2.6)	25 (1.9)
	*Staphylococcus aureus*	33 (1.2)	11 (1.8)	6 (0.7)	16 (1.2)
	*Enterococcus* species^g^	110 (3.9)	20 (3.3)	38 (4.1)	52 (3.9)
	*Aerococcus urinae*	10 (0.4)	^e^	^e^	^e^
	Haemolytic streptococci group A/C/G	5 (0.2)	^e^	^e^	^e^
Tertiary (group III) and normal flora (group IV)	All	23 (0.8)	^e^	^e^	^e^
	Coagulase-negative staphylococci	12 (0.4)	^e^	^e^	^e^
	Other	11 (0.4)	^e^	^e^	^e^

^a^The catchment population increased from approximately 494,000 in the year 2000 to approximately 590,000 in the year 2018

^b^Not including strains identified as extended-spectrum β-lactamase-producing

^c^Strains identified as extended-spectrum β-lactamase-producing

^d^*Salmonella* species and *Staphylococcus saprophyticus* were collapsed due to small cell counts, i.e., ≤5 *Salmonella* species isolated during the entire study period

^e^Collapsed to the species level due to small cell counts

^f^Including *Providencia* species and *Raoultella* species

^g^*Enterococcus* species were classified as positive only when isolated from urine cultures without a group I or II uropathogen in significant concentration, or when the same species was identified in blood culture. Most isolates were genus unspecified; seven were identified as *Enterococcus faecalis*, all accompanied by concordant blood cultures

*ESBL* Extended-spectrum β-lactamase

**Fig. 3:**
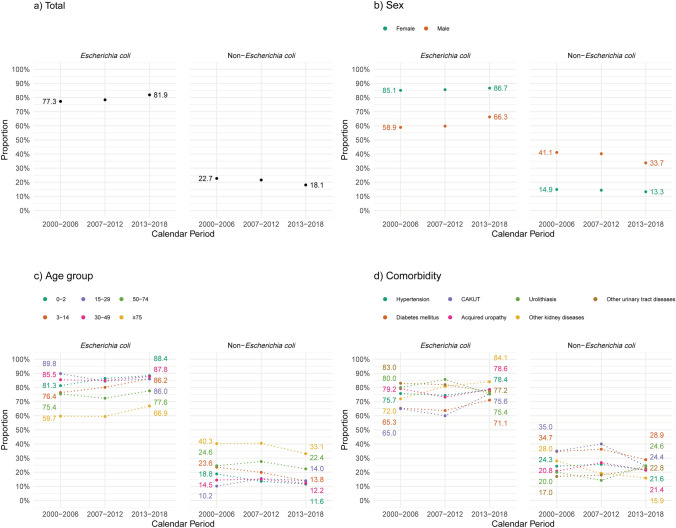
Proportion of APN episodes with *Escherichia coli* and non-*Escherichia coli* species over time^a^, total (**a**) and stratified by sex (**b**), age group (**c**^b^), and comorbidity in age ≥15 years (**d**^b,c^). ^a^ Proportion calculated by dividing the number of isolates of the species during a calendar period (presented in Table 3) by the total number of isolates during the same calendar period (presented in Table 3). ^b^ Dotted lines serve as visual aid and do not represent interpolated data. Some data points overlap and may therefore not be visually distinguishable. ^c^ To maintain consistency with masking of small cell counts in Table 4, the urinary tract cancer stratum was omitted from the figure and legend. *CAKUT* congenital anomalies of the kidney and urinary tract

Over time, the proportion of *E. coli* isolates increased modestly across both sexes and most age groups, rising from 77.3% in 2000-2006 to 81.9% in 2013–2018 (Fig. 3a-c), with only minor fluctuations in the prevalence of individual non-*E. coli* species. The prevalence of ESBL-producing bacteria was low, covering 54 *E. coli* and 5 *K. pneumoniae* isolates, yet the proportion of ESBL-producing *E. coli* rose from 1.0% in 2007-2012 to 3.4% in 2013-2018 (Table 3).

The distribution of pathogens varied by patient characteristics (Table 4 and Fig. 3), with non-*E. coli* uropathogens more commonly detected in male (37.7%) compared with female (13.9%) patients and in those 50 years or older (range: 24.4% to 37.3%) compared with those younger (range: 13.6% to 18.2%). *Klebsiella* species and other non-*E. coli* uropathogens were also more prevalent among patients with diabetes or CAKUT (31.9% in each) than in those without these conditions (Table 4 and Supplementary Table 5).

**Table 4:** Baseline characteristics of patients with hospital-diagnosed acute pyelonephritis and positive urine culture, by microbial species and calendar period

	*Escherichia coli*, n (%)				Non-*Escherichia coli* spp., n (%)			
	Overall	Calendar period^a^			Overall	Calendar period^a^		
		2000-2006	2007-2012	2013-2018		2000-2006	2007-2012	2013-2018
Total	2274 (100.0)	471 (100.0)	719 (100.0)	1084 (100.0)	575 (100.0)	138 (100.0)	198 (100.0)	239 (100.0)
Sex								
Female	1808 (79.5)	365 (77.5)	566 (78.7)	877 (80.9)	293 (51.0)	64 (46.4)	95 (48.0)	134 (56.1)
Male	466 (20.5)	106 (22.5)	153 (21.3)	207 (19.1)	282 (49.0)	74 (53.6)	103 (52.0)	105 (43.9)
Age in years								
Median (IQR)	29 (7-59)	35 (12-65)	22 (4-55)	29 (8-60)	57 (18-75)	62 (20-78)	54 (11-74)	56 (20-74)
0-2	401 (17.6)	65 (13.8)	153 (21.3)	183 (16.9)	63 (11.0)	15 (10.9)	24 (12.1)	24 (10.0)
3-14	297 (13.1)	55 (11.7)	117 (16.3)	125 (11.5)	66 (11.5)	17 (12.3)	29 (14.6)	20 (8.4)
15-29	459 (20.2)	88 (18.7)	131 (18.2)	240 (22.1)	73 (12.7)	10 (7.2)	24 (12.1)	39 (16.3)
30-49	360 (15.8)	94 (20.0)	107 (14.9)	159 (14.7)	57 (9.9)	16 (11.6)	19 (9.6)	22 (9.2)
50-74	493 (21.7)	98 (20.8)	139 (19.3)	256 (23.6)	159 (27.7)	32 (23.2)	53 (26.8)	74 (31.0)
≥75	264 (11.6)	71 (15.1)	72 (10.0)	121 (11.2)	157 (27.3)	48 (34.8)	49 (24.7)	60 (25.1)
Comorbidity in age <15 years								
CAKUT	≤20	^b^	^b^	^b^	≤5	^b^	^b^	^b^
Comorbidity in age ≥15 years								
Hypertension	331 (21.0)	84 (23.9)	87 (19.4)	160 (20.6)	101 (22.6)	27 (25.5)	30 (20.7)	44 (22.6)
Diabetes mellitus	243 (15.4)	47 (13.4)	58 (12.9)	138 (17.8)	114 (25.6)	25 (23.6)	33 (22.8)	56 (28.7)
Uropathies								
CAKUT	62 (3.9)	13 (3.7)	18 (4.0)	31 (4.0)	29 (6.5)	7 (6.6)	12 (8.3)	10 (5.1)
Acquired obstructive and reflux uropathy	104 (6.6)	19 (5.4)	30 (6.7)	55 (7.1)	31 (7.0)	5 (4.7)	11 (7.6)	15 (7.7)
Urolithiasis	93 (5.9)	20 (5.7)	30 (6.7)	43 (5.5)	24 (5.4)	5 (4.7)	5 (3.4)	14 (7.2)
Urinary tract cancer	≤24	^b^	^b^	^b^	≤5	^b^	^b^	^b^
Other kidney disease	76 (4.8)	18 (5.1)	21 (4.7)	37 (4.8)	19 (4.3)	7 (6.6)	5 (3.4)	7 (3.6)
Other urinary tract disease	172 (10.9)	44 (12.5)	50 (11.1)	78 (10.1)	43 (9.6)	9 (8.5)	11 (7.6)	23 (11.8)

^a^The catchment population increased from approximately 494,000 in the year 2000 to approximately 590,000 in the year 2018

^b^Not stratified due to small cell counts

*CAKUT* congenital anomalies of the kidney and urinary tract; *IQR* interquartile range; *spp* species

### Clinical outcomes

Clinical outcomes varied by causative pathogen (Table 5 and Supplementary Table 6). Overall, LOS decreased by 2 days over the study period and remained shorter for patients with *E. coli* APN (median 4 days [IQR: 2-6, 95% CI 4-4]) compared with non-*E. coli* APN (median 5 days [IQR: 3-8, 95% CI 4-5]). The longest LOS was observed in patients with *Pseudomonas aeruginosa* APN, though this decreased from a median of 8 days (IQR: 4-12, 95% CI 4-12) in earlier years to 4 days (IQR: 2-7, 95% CI 3-7) in later years. Thirty-day mortality was also higher among patients with non-*E. coli* APN (cumulative mortality 0.037 [95% CI 0.023-0.052]) compared with *E. coli* APN (cumulative mortality 0.010 [95% CI 0.006-0.015]). ESBL-producing *E. coli* APN showed similar LOS and mortality to *E. coli* APN overall.

**Table 5:** Clinical outcomes of patients with hospital-diagnosed acute pyelonephritis and positive urine culture, by microbial species and calendar period

	All isolates	*Escherichia coli*				Non-*Escherichia coli* spp.			
	Overall	Overall	Calendar period^a^			Overall	Calendar period^a^		
			2000-2006	2007-2012	2013-2018		2000-2006	2007-2012	2013-2018
Total, n (%)	2849 (100.0)	2274 (100.0)	471 (100.0)	719 (100.0)	1084 (100.0)	575 (100.0)	138 (100.0)	198 (100.0)	239 (100.0)
Median LOS, days (IQR) [95% CI]	4 (2-6) [4-4]	4 (2-6) [4-4]	5 (4-8) [5-5]	4 (3-6) [4, 5]	3 (1-4) [3-3]	5 (3-8) [4, 5]	6 (3-9) [5, 6]	5 (3-9) [5, 6]	4 (1-6) [3, 4]
Died within 30 days, n (%)	44 (1.5)	23 (1.0)	≤10	≤5	12 (1.1)	21 (3.7)	≤5	≤15	8 (3.3)
30-day cumulative mortality (95% CI)	0.015 (0.011-0.020)	0.010 (0.006-0.015)	^b^	^b^	^b^	0.037 (0.023-0.052)	^b^	^b^	^b^

^a^The catchment population increased from approximately 494,000 in the year 2000 to approximately 590,000 in the year 2018

^b^Not stratified due to small cell counts

*CI* confidence interval; *ESBL* extended-spectrum β-lactamase; *IQR* interquartile range; *LOS* length of stay; *spp* species

### Sensitivity analyses

Raising the cut-off concentration defining a positive urine culture to ≥10^5^ CFU/mL reduced the number of urine culture-positive episodes by 216, corresponding to a 4-percentage point reduction compared with the primary analysis (Supplementary Table 7). The pathogen distribution across 2621 isolates remained largely unchanged, with *E. coli* accounting for 78.1%, and temporal trends comparable to those observed in the primary analysis.

Expanding the urine culture inclusion time window to span an additional 9 and 25 days, respectively, had minimal effect on culture capture, identifying merely 70 and 103 additional episodes (Supplementary Table 7). However, we noted minor shifts in pathogen distribution, particularly an increase in *Enterococcus* species, rising from 3.9% in the primary analysis to 6.9% and 7.2%, respectively.

## Discussion

In this 19-year population-based cohort study, we identified an increasing use of microbiological diagnostics in the evaluation of patients with hospital-diagnosed APN. *E. coli* remained the dominant uropathogen, followed by *K. pneumoniae*, with only modest changes in pathogen distribution over time. The proportion of *E. coli* increased across most age groups, while non-*E. coli* uropathogens declined slightly overall but remained more common in male patients, older adults, and patients with diabetes or CAKUT. Although the overall LOS decreased during the study period, patients with non-*E. coli* APN consistently exhibited slightly longer hospital stay and higher 30-day mortality compared with *E. coli* APN.

### Interpretation and comparison with existing literature

In light of the recently reported rise in APN incidence in Denmark, our observations likely reflect a shift in diagnostic practice, where more comprehensive testing has facilitated more anatomically specific infection diagnoses, such as distinguishing APN from unspecified UTIs [3]. While demographic factors, including population ageing and a growing burden of diabetes and urolithiasis among patients with APN [3], may have increased susceptibility to non- *E. coli* infections [12, 20], such concurrent changes in diagnostic practice may have contributed to more frequent classification of milder *E. coli* infections as APN. Together, these trends may help explain the relatively stable pathogen distribution observed over time.

Globally, the uropathogen distribution shows substantial variation by geographical and clinical setting [21, 22]. *E. coli* accounted for nearly 80% of cases in our study, markedly higher than the 45% reported among urological patients with APN by a global review [23]. However, our findings align with a Canadian study, identifying *E. coli* in approximately 80% of all female in- and outpatient APN cases [19], and with studies showing higher proportions of non-*E. coli* pathogens and ESBL-producing *E. coli* in men and older adults [19, 24, 25]. This distribution may be explained by the higher prevalence of risk factors, such as urinary flow obstruction (*e.g.*, due to prostate hypertrophy), and an increased risk of healthcare-associated UTIs (*e.g.*, due to indwelling urinary catheters) in these patient groups [5, 11, 26]. We also observed a higher prevalence of non-*E. coli* pathogens, particularly *Klebsiella* species, in patients with diabetes or CAKUT. While diabetes is not typically associated with specific bacterial uropathogens, similar distributions of *Klebsiella* species in diabetic patients have been reported [20]. Likewise, a high prevalence of *Klebsiella* infections in children with CAKUT has previously been documented [27]. These patterns may partly be attributable to recurrent UTIs and repeated antibiotic use in these populations, potentially selecting more resistant and less common pathogens. In the present study, *Enterococcus* species accounted for a small proportion of isolates and were classified as causative pathogens only in the absence of primary or secondary uropathogens, or when supported by concordant blood cultures. This finding underscores the importance of cautious interpretation of enterococcal growth in urine cultures [28], while recognising that enterococci may occasionally represent causative pathogens in patients with hospital-diagnosed APN.

Several previous studies on so-called complicated UTIs have reported in-hospital and 30-day mortality ranging from 0.1% to 8.7%, with mortality among patients with APN at the lower end of this range [1, 2, 11, 26, 29–31]. Consistent with our findings, the few of these that examined mortality by microbial aetiology, reported higher mortality associated with non-*E. coli* pathogens [29, 31]. For *Klebsiella* species, mortality ranged from 10.3% to 30.8%, compared with 5.8% to 28.1% for *E. coli.* Similarly, reported LOS for complicated UTIs vary widely, from 4 to 27 days across different populations [1, 2, 11, 26, 29–31]. However, to our knowledge, no prior study has evaluated LOS in APN by the causative pathogen. Thus, our finding of a modestly longer LOS in patients with non-*E. coli* APN compared with those with *E. coli* is novel. In comparing outcomes of ESBL- and non-ESBL-related infections, our finding that patients with ESBL-producing *E. coli* APN did not demonstrate worse clinical outcomes contrasts with a recent meta-analysis [32]. In our setting, this might be explained by the continued susceptibility of ESBL strains to mecillinam, which was commonly used in empirical treatment in Nordic countries [33], potentially improving early treatment adequacy.

### Strengths and limitations

This study’s strengths include its population- and register-based design, which enabled the examination of long-term trends in microbiological diagnostics and microbial aetiology among patients with hospital-diagnosed APN. The inclusion of patients across all age groups and linkage of national registries with microbiological data allowed for robust characterisation of patient demographics and comorbidities in relation to microbial findings. Given that the study population comprised both urban and rural residents and represented about 10% of Denmark’s population, the findings are likely generalisable to the broader Danish context and potentially to other Nordic countries with similar healthcare systems, demographics, and low antimicrobial resistance rates.

This study lacked access to clinical records, limiting diagnostic validation of APN symptoms. Prior studies have estimated a positive predictive value of 76% to 80% for APN diagnoses in the Danish National Patient Registry, and some degree of misclassification, *e.g.*, from inclusion of non-infectious nephritis, is possible [3]. Considering that one-third of the included patients had received outpatient antibiotic therapy prior to urine sampling, the relatively high proportion of urine culture positivity, particularly in the later study years, may indicate reasonable diagnostic validity. Conversely, less frequent urine culturing in the earlier years may have led to underdiagnosis and selection bias. In addition, lack of data on hospital-administered antibiotics and inconsistent recording of catheterisation procedures limited assessment of prior antimicrobial exposure and key risk factors. To enhance identification of clinically relevant isolates, especially in patients pre-treated with antibiotics, we applied a low cut-off concentration of ≥10^3^ CFU/mL with AST in the primary analysis [16]. While this may have reduced specificity by including some contaminants, results were consistent in the sensitivity analysis using the higher ≥10^5^ CFU/mL cut-off.

## Conclusion

Over the 19-year study period, the use of microbiological diagnostics among patients presenting to hospital with APN increased, whereas the pathogen distribution remained largely stable, with *E. coli* as the predominant uropathogen. Non-*E. coli* infections were more common in older, male, and comorbid patients and were associated with slightly less favourable short-term outcomes. These findings provide updated microbiological and clinical insight into APN epidemiology.

## Supplementary Information

Below is the link to the electronic supplementary material.

Supplementary file1 (PDF 475 KB)

## Data Availability

Data generated in this study are included in this published article and its supplementary information file. The underlying individual-level data used in this study are not publicly available and cannot be made publicly available by the authors due to Danish legal restrictions. Researchers can apply for access to Danish healthcare data through the Danish Health Data Authority (https://sundhedsdatastyrelsen.dk/da/english). Access to microbiological data can be requested via the relevant Regional Council (https://stps.dk/sundhedsfaglig/ansvar-og-retningslinjer/patientjournaloplysninger-til-forskning in Danish) or through the Statens Serum Institut (https://miba.ssi.dk/forskningsbetjening/ansoeg-om-data-via-dias, in Danish).
