# Supplementary material for: Uropathogens and prognosis among patients with hospital-diagnosed acute pyelonephritis: insights from a 19-year population-based cohort study
Source: Infection. 2026 Jan 23;54(2):917–29. doi: 10.1007/s15010-026-02729-7 (PMC13021717; doi:10.1007/s15010-026-02729-7)
Supplement: Supplementary file 1 — Supplementary file1 (PDF 475 KB) [file 15010_2026_2729_MOESM1_ESM.pdf]

Infection

Supplementary Information

Uropathogens and prognosis in severe acute pyelonephritis: insights from a 19-year population-based cohort study

Lise Skovgaard Svingel*, Mette Nørgaard, Christian Fynbo Christiansen, Henrik Birn, Hans Linde Nielsen, Kirstine Kobberøe Søgaard

***Corresponding author:** Lise Skovgaard Svingel; Department of Clinical Epidemiology, Department of Clinical Medicine, Aarhus University Hospital and Aarhus University, Olof Palmes Allé 43-45, 8200 Aarhus N, Denmark; lisskg@clin.au.dk

Contents

Supplementary tables 1

- **Supplementary Table 1** Description of the data sources used in this study 1
- **Supplementary Table 2** Codes used for inclusion, exclusion, and patient characterization 2
- **Supplementary Table 3** Categorization of uropathogens and classification of the result of urine culture in the primary analysis 5
- **Supplementary Table 4** Baseline characteristics of patients with non-urine culture-confirmed, hospital-diagnosed acute pyelonephritis, stratified by test characteristics and calendar period 7
- **Supplementary Table 5** Baseline characteristics of patients with hospital-diagnosed acute pyelonephritis and positive urine culture, by microbial species and calendar period 10
- **Supplementary Table 6** Clinical outcomes of patients with hospital-diagnosed acute pyelonephritis and positive urine culture, by microbial species and calendar period 12
- **Supplementary Table 7** Number of episodes and patients with urine cultures identified by application of different time windows for identification of urine cultures related to acute pyelonephritis diagnosis and cut-off concentration 13

References 14

Supplementary tables

Supplementary Table 1 Description of the data sources used in this study

The Danish Civil Registration System [1]**:** This registry contains individual-level data on all Danish residents, including date of birth, sex, residency, migration, and vital status. Each resident is assigned a unique 10-digit personal identifier used across all public registries, enabling accurate data linkage across all data sources and virtually complete follow-up.

The Danish National Patient Registry (DNPR) [2]: This registry includes data on all somatic hospital admissions since 1977 and emergency and outpatient visits since 1995, including on diseases and surgical procedures. Diagnoses are coded following the International Classification of Diseases, 8th Revision (ICD-8) until 1994 and 10th Revision (ICD-10) thereafter.

The Danish National Prescription Registry [3]: This registry contains data on all prescriptions, including on antibiotics prescribed for outpatient treatment of UTIs, dispensed at community pharmacies since 1995.

wwLab/ADBakt (Autonik AB, Nyköping, Sweden) [4]: The laboratory information system recording data on all urine and blood cultures performed at the Department of Clinical Microbiology, Aalborg University Hospital. Urine culture procedures followed European Urinalysis Guidelines [5], and blood culturing was performed using automated systems [4,6]. Bacterial identification was performed using conventional biochemical diagnostic methods and MALDI-TOF (Bruker, Bremen, Germany) [4]. Antimicrobial susceptibility testing followed the Swedish Reference Group of Antibiotics guidelines until 2010 [7], and European Committee of Antimicrobial Susceptibility Testing (EUCAST) guidelines thereafter [8]. Detection of Extended-spectrum β-lactamase (ESBL)-producing *E. coli* and *Klebsiella pneumoniae* followed previously published methods for this setting [9].

Supplementary Table 2 Codes used for inclusion, exclusion, and patient characterization

Disorder	Data source	ICD-8 code	ICD-10 code	ATC code	Description	Specifications for analysis
**Diagnoses for inclusion**						
Acute pyelonephritis	DNPR [2]		N109 (year 1994-2011)		Acute tubulo-interstitial nephritis, NOS	Primary and secondary discharge diagnoses; by admission date for inpatient admissions, emergency department/medical admission unit visits.
			N109C (year 2012-)		Acute pyelonephritis	
			N118B (year 2012-)		Recurrent pyelonephritis	
			N129		Pyelonephritis, NOS	
			N209A		Calculous pyelonephritis	
			O230		Pyelonephritis in pregnancy	
			O862B		Pyelonephritis following delivery	
			P393		Neonatal urinary tract infection	
**Diagnoses for exclusion**						
Chronic pyelonephritis	DNPR [2]		N11 (without N118)		Chronic tubulo-interstitial nephritis	Primary and secondary discharge diagnoses; by discharge date for inpatient admissions and emergency department/medical admission unit visits.
			N200I, N201I, N202I		Infectious calculus of kidney and/or ureter	
**Diagnoses for characterization**						
Hypertension	DNPR [2]	400-404	I10-I15 (without I120, I131, I132)		Hospital-diagnosed hypertension	Primary and secondary discharge diagnoses; by discharge date for inpatient admissions and outpatient clinic visits.
	The Danish National Prescription Registry [3]			C02A, C02C	α-adrenergic blockers	Combination treatment of at least two redeemed prescriptions for different types of the following classes of antihypertensive drugs within 180 days prior to the index date; by date of filled prescription.
				C02DA, C02L, C03A, C03B, C03D, C03E, C03X, C07C, C07D, C08G, C09BA, C09DA, C09XA52, C09DX01	Non-loop diuretics	
				C02DB, C02DD, C02DG, C04, C05	Vasodilators	
				C07	β-blockers	
				C07F, C08, C09BB, C09DB, C09DX01	Calcium channel blockers	
				C09	Renin-angiotensin system inhibitors	
Diabetes mellitus	DNPR [2]	249.00, 249.06, 249.07,	E10-E14		Diabetes mellitus	Primary and secondary discharge diagnoses;
			O24 (without O24.4)		Diabetes mellitus in pregnancy (except arising in pregnancy)	

		249.09, 250.00, 250.06, 250.07, 250.09	G632		Diabetic polyneuropathy	by discharge date for inpatient admissions and outpatient clinic visits.
			H360		Diabetic retinopathy	
			N083		Glomerular disorders in diabetes mellitus	
	The Danish National Prescription Registry [3]			A10A, A10B	All antidiabetic medication (insulin, metformin, sulfonylureas, any other antidiabetic drugs)	By date of filled prescription.
Congenital anomalies of the kidney and urinary tract (CAKUT)	DNPR [2]	753	Q60		Kidney agenesis and other reduction defects of kidney	Primary and secondary discharge diagnoses; by discharge date for inpatient admissions and outpatient clinic visits.
			Q61		Cystic kidney disease	
			Q62		Congenital obstructive defects of kidney pelvis and congenital malformations of ureter	
			Q63		Other congenital malformations of kidney	
			Q64		Other congenital malformations of urinary system	
Acquired obstructive and reflux uropathy	DNPR [2]	591, 593.30-50, 593.52, 598	N13, N320, N35, N991		Obstructive and reflux uropathy incl. stricture of urinary structures	Primary and secondary discharge diagnoses; by discharge date for inpatient admissions and outpatient clinic visits.
Urolithiasis	DNPR [2]	592.00-592.03, 592.08-592.09, 594	N20-22 (without N200I, N201I, N202I)		Calculus of the urinary system (non-infectious)	Primary and secondary discharge diagnoses; by discharge date for inpatient admissions and outpatient clinic visits.
Urinary tract cancer	DNPR [2]	188, 189	C64-C68		Malignant neoplasm of urinary organs (without carcinoma in situ)	Primary and secondary discharge diagnoses; by discharge date for inpatient admissions and outpatient clinic visits.
Other kidney diseases	DNPR [2]	580, 582, 583	N00, N01, N03, N05		Glomerulonephritis (without nephrotic syndrome)	Primary and secondary discharge diagnoses; by discharge date for inpatient admissions and outpatient clinic visits.
		581	N04		Nephrotic syndrome	
		403, 404	I12, I13, I150, I151		Hypertensive kidney disease without kidney failure	
		593.20	N14, N15, N16		Interstitial nephritis	
		249.02, 250.02	E102, E112, E142, N083		Diabetic nephropathy	
		584, 792	N07-08, N18-19, N26-27		Other and unknown chronic kidney disease	
Other urinary tract diseases	DNPR [2]	N596, N597, 599	N31-33 (without N330 and N320), N36-39 (without N370 and N390)		Other diseases of the urinary system	Primary and secondary discharge diagnoses; by discharge date for inpatient admissions and outpatient clinic visits.

Interventional urological procedures	DNPR [2]	KK, KTK, KUK			Primary and secondary diagnoses;<br>by date of procedure/surgery.
Antibiotic treatment of UTI	The Danish National Prescription Registry [3]		J01CA02,<br>J01CA04,<br>J01CA08,<br>J01EA01,<br>J01EB02,<br>J01CR02,<br>J01MA02,<br>J01XE01	Pivampicillin,<br>Amoxicillin,<br>Pivmecillinam,<br>Trimethoprim,<br>Sulfamethizol,<br>Amoxicillin with beta-lactamase inhibitor,<br>Ciprofloxacin,<br>Nitrofurantoin	By date of filled prescription.
Urinary tract imaging	DNPR [2]	UXCD62, UXMD62, UXRD65;<br>UXUD61, UXUD75;<br>WNCG211XX, WNCGD11A1, WNCGD11F2, WNCGD11NG, WNCGD11XX;<br>WNCGS14XX		Urography (computerized tomography, magnetic resonance, intravenous);<br>Kidney and bladder ultrasound;<br>Renography (technetium-99m-MAG3);<br>Renal Scintigraphy (technetium-99m-DMSA)	Primary and secondary diagnoses;<br>by procedure date.

Abbreviations: DNPR: Danish National Patient Registry; ICD-8: International Classification of Diseases, 8th Revision; ICD-10: International Classification of Diseases, 10th Revision; NOS: not otherwise specified; UTI: urinary tract infection.

Supplementary Table 3 Categorization of uropathogens and classification of the result of urine culture in the primary analysis

Species	Pathogenicity in the urinary tract[a]	Cut-off bacterial concentration (CFU/mL)[b]	Additional requirements	Urine culture classified as positive[c]
Escherichia coli<br>*Salmonella* spp.<br>*Staphylococcus saprophyticus*	Primary pathogens	≥10^3	One or two species isolates[d] and accompanying AST	Yes
Klebsiella spp.<br>*Citrobacter* spp.<br>*Enterobacter* spp.<br>*Proteus* spp.<br>*Serratia* spp.<br>*Morganella morganii*<br>*Providencia* spp.<br>*Raoultella* spp.<br>*Pseudomonas aeruginosa*<br>*Haemophilus* spp.<br>*Staphylococcus aureus*<br>*Enterococcus* spp.[e]<br>*Aerococcus* spp.<br>Haemolytic streptococci (except group B[f])	Secondary pathogens	≥10^3	One or two species isolates[d] and accompanying AST	Yes

Pseudomonas spp. (except *P. aeruginosa*)<br><br>*Acinetobacter* spp.<br><br>*Stenotrophomonas maltophilia*<br><br>*Candida* spp.<br><br>Coagulase-negative staphylococci (except *S. saprophyticus*)<br><br>Group B streptococci[f]	Tertiary pathogens	$\geq 10^3$	One species isolated and<br><br>accompanying AST	Yes
Non-haemolytic streptococci<br><br>Other normal urogenital flora	Normal flora	Any	Same species was identified in blood culture and<br><br>accompanying AST	Yes

[a] As defined by European Urinalysis Guidelines [5]
[b] In urine collected from mid-stream urine, indwelling catheter, suprapubic aspiration, cystoscopy, or single urethral catheterization.
[c] Urine containing any concentration of group I-IV species was considered positive if the same species was identified in blood culture.
[d] Urine was considered contaminated when more than two species were present.
[e] *Enterococcus* species were classified as positive only when isolated from urine cultures without a group I or II uropathogen in significant concentration, or when the same species was identified in blood culture.
[f] Group B streptococci were classified as group II uropathogen in female patients aged 15-40 years (considered reproductive age).
Abbreviations: AST: antimicrobial susceptibility testing; CFU: colony forming units; spp: species.

Supplementary Table 4 Baseline characteristics of patients with non-urine culture-confirmed, hospital-diagnosed acute pyelonephritis, stratified by test characteristics and calendar period

	Episodes without urine culture, n (%)				Episodes with non-positive urine culture[a], n (%)			
	Overall	Calendar period[b]			Overall	Calendar period[b]		
		2000-2006	2007-2012	2013-2018		2000-2006	2007-2012	2013-2018
**Total**	**786 (100.0)**	**331 (100.0)**	**294 (100.0)**	**161 (100.0)**	**1,779 (100.0)**	**408 (100.0)**	**551 (100.0)**	**820 (100.0)**
**Sex**								
Female	555 (70.6)	229 (69.2)	206 (70.1)	120 (74.5)	1,296 (72.8)	290 (71.1)	415 (75.3)	591 (72.1)
Male	231 (29.4)	102 (30.8)	88 (29.9)	41 (25.5)	483 (27.2)	118 (28.9)	136 (24.7)	229 (27.9)
**Age in years**								
Median (IQR)	49 (25-74)	55 (26-77)	45 (25-73)	44 (23-72)	42 (22-66)	40 (23-66)	39 (20-64)	45 (25-67)
0-2	27 (3.4)	8 (2.4)	13 (4.4)	6 (3.7)	80 (4.5)	21 (5.1)	31 (5.6)	28 (3.4)
3-14	31 (3.9)	13 (3.9)	8 (2.7)	10 (6.2)	150 (8.4)	42 (10.3)	53 (9.6)	55 (6.7)
15-29	195 (24.8)	82 (24.8)	71 (24.1)	42 (26.1)	403 (22.7)	87 (21.3)	131 (23.8)	185 (22.6)
30-49	144 (18.3)	51 (15.4)	66 (22.4)	27 (16.8)	380 (21.4)	91 (22.3)	110 (20.0)	179 (21.8)
50-74	194 (24.7)	81 (24.5)	72 (24.5)	41 (25.5)	498 (28.0)	95 (23.3)	152 (27.6)	251 (30.6)
≥75	195 (24.8)	96 (29.0)	64 (21.8)	35 (21.7)	268 (15.1)	72 (17.6)	74 (13.4)	122 (14.9)
**Comorbidity in age <15 years**								
CAKUT	≤5	[c]	[c]	[c]	≤5	[c]	[c]	[c]

	Episodes without urine culture, n (%)				Episodes with non-positive urine culture[a], n (%)			
	Overall	Calendar period[b]			Overall	Calendar period[b]		
		2000-2006	2007-2012	2013-2018		2000-2006	2007-2012	2013-2018
**Comorbidity in age ≥15 years**								
Hypertension	160 (22.0)	73 (23.5)	62 (22.7)	25 (17.2)	349 (22.5)	72 (20.9)	112 (24.0)	165 (22.4)
Diabetes mellitus	116 (15.9)	41 (13.2)	50 (18.3)	25 (17.2)	230 (14.8)	47 (13.6)	66 (14.1)	117 (15.9)
Uropathies								
CAKUT	23 (3.2)	[c]	[c]	[c]	55 (3.6)	10 (2.9)	19 (4.1)	26 (3.5)
Acquired obstructive and reflux uropathy	55 (7.6)	29 (9.4)	16 (5.9)	10 (6.9)	114 (7.4)	28 (8.1)	40 (8.6)	46 (6.2)
Urolithiasis	40 (5.5)	17 (5.5)	15 (5.5)	8 (5.5)	88 (5.7)	17 (4.9)	32 (6.9)	39 (5.3)
Urinary tract cancer	15 (2.1)	≤5	7 (2.6)	≤5	26 (1.7)	6 (1.7)	7 (1.5)	13 (1.8)
Other kidney diseases	34 (4.7)	16 (5.2)	10 (3.7)	8 (5.5)	79 (5.1)	18 (5.2)	25 (5.4)	36 (4.9)
Other urinary tract diseases	74 (10.2)	29 (9.4)	25 (9.2)	20 (13.8)	161 (10.4)	38 (11.0)	44 (9.4)	79 (10.7)
**Recent events[d]**								
Inpatient admission	85 (25.7)	87 (29.6)	39 (24.2)	85 (25.7)	432 (24.3)	97 (23.8)	134 (24.3)	201 (24.5)
Urological procedure	11 (3.3)	10 (3.4)	5 (3.1)	11 (3.3)	56 (3.1)	12 (2.9)	16 (2.9)	28 (3.4)
Antibiotics	103 (31.1)	93 (31.6)	36 (22.4)	103 (31.1)	798 (44.9)	192 (47.1)	248 (45.0)	358 (43.7)
**Additional tests[e]**								
Blood culture	268 (34.1)	71 (21.5)	106 (36.1)	91 (56.5)	1,422 (79.9)	310 (76.0)	434 (78.8)	678 (82.7)

	Episodes without urine culture, n (%)				Episodes with non-positive urine culture[a], n (%)			
	Overall	Calendar period[b]			Overall	Calendar period[b]		
		2000-2006	2007-2012	2013-2018		2000-2006	2007-2012	2013-2018
Imaging	613 (78.0)	258 (77.9)	229 (77.9)	126 (78.3)	1,390 (78.1)	312 (76.5)	439 (79.7)	639 (77.9)

[a] Urine cultures were classified as non-positive if the specimen was collected using an unreliable method (*e.g.*, bottle or bedpan), the bacterial concentration was below the predefined cut-off, or the culture was deemed contaminated (*i.e.*, containing more than two species and not accompanied by a positive blood culture identifying the same species).

[b] The catchment population increased from approximately 494,000 in the year 2000 to approximately 590,000 in the year 2018.

[c] Not stratified due to small cell counts.

[d] Within 30 days prior to the index date.

[e] From 7 days before to 2 days after the index date.

Abbreviations: CAKUT: congenital anomalies of the kidney and urinary tract; IQR, interquartile range.

Supplementary Table 5 Baseline characteristics of patients with hospital-diagnosed acute pyelonephritis and positive urine culture, by microbial species and calendar period

	**ESBL-*Escherichia coli*, n (%)**	***Klebsiella* spp., n (%)**	***Pseudomonas aeruginosa*, n (%)**	***Enterococcus* spp.[a], n (%)**
	**Overall**	**Overall**	**Overall**	**Overall**
**Total**	54 (100.0)	187 (100.0)	69 (100.0)	110 (100.0)
**Sex**				
Female	35 (64.8)	98 (52.4)	30 (43.5)	56 (50.9)
Male	19 (35.2)	89 (47.6)	39 (56.5)	54 (49.1)
**Age in years**				
Median (IQR)	40 (20-74)	57 (18-76)	62 (11-77)	37 (7-76)
0-2	5 (9.3)	25 (13.4)	≤10	17 (15.5)
3-14	5 (9.3)	15 (8.0)	13 (18.8)	17 (15.5)
15-29	12 (22.2)	22 (11.8)	≤10	16 (14.5, 3.0)
30-49	7 (13.0)	17 (9.1)	6 (8.7)	9 (8.2)
50-74	12 (22.2)	57 (30.5)	20 (29.0)	18 (16.4)
≥75	13 (24.1)	51 (27.3)	23 (33.3)	33 (30.0)
**Comorbidity in age <15 years**				
CAKUT	≤5	≤5	0 (0.0)	≤5
**Comorbidity in age ≥15 years**				
Hypertension	5 (11.4)	30 (20.4)	14 (27.5)	20 (26.3)

	ESBL-*Escherichia coli*, n (%)	*Klebsiella* spp., n (%)	*Pseudomonas aeruginosa*, n (%)	*Enterococcus* spp.[a], n (%)
	**Overall**	**Overall**	**Overall**	**Overall**
Diabetes mellitus	8 (18.2)	45 (30.6)	10 (19.6)	23 (30.3)
Uropathies				
CAKUT	(0.0)	10 (6.8)	≤5	≤5
Acquired obstructive and reflux uropathy	≤5	11 (7.5)	5 (9.8)	5 (6.6)
Urolithiasis	≤5	6 (4.1)	≤5	7 (9.2)
Urinary tract cancer	≤5	≤5	(0.0)	≤5
Other kidney diseases	≤5	7 (4.8)	(0.0)	6 (7.9)
Other urinary tract diseases	≤5	15 (10.2)	5 (9.8)	15 (19.7)

[a] *Enterococcus* species were classified as positive only when isolated from urine cultures without a group I or II uropathogen in significant concentration, or when the same species was identified in blood culture. Most isolates were genus unspecified; seven were identified as *Enterococcus faecalis*, all accompanied by concordant blood cultures.
Abbreviations: CAKUT: congenital anomalies of the kidney and urinary tract; IQR, interquartile range; spp: species.

Supplementary Table 6 Clinical outcomes of patients with hospital-diagnosed acute pyelonephritis and positive urine culture, by microbial species and calendar period

	ESBL-*Escherichia coli*				*Klebsiella* spp.				*Pseudomonas aeruginosa*				*Enterococcus* spp.[a]			
	Overall	Calendar period[b]			Overall	Calendar period[b]			Overall	Calendar period[b]			Overall	Calendar period[b]		
		2000-2006	2007-2012	2013-2018		2000-2006	2007-2012	2013-2018		2000-2006	2007-2012	2013-2018		2000-2006	2007-2012	2013-2018
**Total, n (%)**	**54 (100.0)**	**0 (0.0)**	**9 (100.0)**	**45 (100.0)**	**187 (100.0)**	**45 (100.0)**	**64 (100.0)**	**78 (100.0)**	**69 (100.0)**	**20 (100.0)**	**24 (100.0)**	**25 (100.0)**	**110 (100.0)**	**20 (100.0)**	**38 (100.0)**	**52 (100.0)**
**Median LOS, days (IQR) [95% CI]**	4 (2-6) [2-5]	NA	4 (3-10) [2-10]	4 (1-5) [2-5]	5 (3-7) [4-5]	6 (5-8) [5-7]	5 (3-7) [4-6]	4 (2-5) [3-5]	7 (4-11) [4-8]	8 (4-12) [4-12]	7 (5-13) [5-13]	4 (2-7) [3-7]	4 (2-8) [3-5]	6 (4-11) [4-11]	5 (3-10) [3-8]	3 (1-6) [2-5]
**Died within 30 days, n (%)**	0 (0.0)	NA	0 (0.0)	0 (0.0)	≤5	c	c	c	≤5	c	c	c	7 (6.4)	c	c	c
**30-day cumulative mortality (95%)**	0 (0-0)	c	c	c	0.011 (0-0.027)	c	c	c	0.014 (0-0.043)	c	c	c	0.064 (0.027-0.109)	c	c	c

[a] *Enterococcus* species were classified as positive only when isolated from urine cultures without a group I or II uropathogen in significant concentration, or when the same species was identified in blood culture. Most isolates were genus unspecified; seven were identified as *Enterococcus faecalis*, all accompanied by concordant blood cultures.

[b] The catchment population increased from approximately 494,000 in the year 2000 to approximately 590,000 in the year 2018.

[c] Not stratified due to small cell counts.

Abbreviations: CI: confidence interval; ESBL: extended-spectrum β-lactamase; IQR: interquartile range; LOS: length of stay; spp: species.

Supplementary Table 7 Number of episodes and patients with urine cultures identified by application of different time windows for identification of urine cultures related to acute pyelonephritis diagnosis and cut-off concentration

		All episodes	Episodes with urine culture			
**Time window**			**From 7 days before to 2 days after index**	**From 7 days before to 2 days after index[a]**	**From 14 days before to 4 days after index**	**From 30 days before to 4 days after index**
**Cut-off concentration**			**≥10^5 CFU/mL**	**≥10^3 CFU/m[a]**	**≥10^3 CFU/mL**	**≥10^3 CFU/mL**
**Total**	Episodes, n (%)	5,338 (100.0)				
	Patients, n (%)	4,773 (100.0)				
**Urine culture performed**	Episodes, n (%)		4,552 (85.3)	4,552 (85.3)	4,622 (86.6)	4,655 (87.2)
	Patients, n (%)		4,099 (85.9)	4,099 (85.9)	4,154 (87.0)	4,173 (87.4)
**Positive urine culture**	Episodes, n (%)		2,557 (47.9)	2,773 (51.9)	2,851 (53.4)	2,926 (54.8)
	Patients, n (%)		2,384 (49.9)	2,563 (53.7)	2,630 (55.2)	2,687 (56.3)
**Concordant urine and blood culture**	Episodes, n (%)		530 (9.9)	530 (9.9)	535 (10.0)	539 (10.1)
	Patients, n (%)		526 (11.0)	526 (11.0)	531 (11.1)	535 (11.2)

[a] Primary analysis.
Abbreviations: CFU: colony forming units.

References

1. Schmidt M, Schmidt SAJ, Adelborg K, Sundbøll J, Laugesen K, Ehrenstein V, et al. The Danish health care system and epidemiological research: from health care contacts to database records. Clin Epidemiol. 2019;11:563–91. https://doi.org/DOI:%2520https://doi.org/10.2147/CLEP.S179083

2. Schmidt M, Schmidt SA, Sandegaard JL, Ehrenstein V, Pedersen L, Sorensen HT. The Danish National Patient Registry: a review of content, data quality, and research potential. Clin Epidemiol. 2015;7:449–90. https://doi.org/DOI:%2520https://doi.org/10.2147/clep.s91125

3. Pottegard A, Schmidt SAJ, Wallach-Kildemoes H, Sorensen HT, Hallas J, Schmidt M. Data Resource Profile: The Danish National Prescription Registry. Int J Epidemiol. 2017;46:798–798f. https://doi.org/10.1093/ije/dyw213

4. Schonheyder HC, Sogaard M. Existing data sources for clinical epidemiology: The North Denmark Bacteremia Research Database. Clin Epidemiol. 2010;2:171–8.

5. European Confederation of Laboratory Medicine EUG. European urinalysis guidelines. Scand J Clin Lab Invest Suppl. 2000;231:1–86.

6. Gradel KO, Schønheyder HC, Arpi M, Knudsen JD, Ostergaard C, Søgaard M. The Danish Collaborative Bacteraemia Network (DACOBAN) database. Clin Epidemiol. 2014;6:301–8. https://doi.org/10.2147/clep.S66998

7. Olsson-Liljequist B, Forsgren A. Antimicrobial susceptibility testing in Sweden. I. The work of the Swedish Reference Group for Antibiotics (SRGA and SRGA-M). Scand J Infect Dis Suppl. 1997;105:5–7.

8. The European Committee on Antimicrobial Susceptibility Testing. Breakpoint tables for interpretation of MICs and zone diameters.

9. Richelsen R, Smit J, Anru PL, Schønheyder HC, Nielsen H. Incidence of community-onset extended-spectrum β-lactamase-producing Escherichia coli and Klebsiella pneumoniae infections: an 11-year population-based study in Denmark. Infect Dis (Lond). 2020;52:547–56. https://doi.org/DOI:%2520https://doi.org/10.1080/23744235.2020.1763452
